# Revision of medial unicompartmental knee arthroplasty—Not as uncomplicated as one thought? Analysis of survival and re‐revisions from a single centre

**DOI:** 10.1002/jeo2.70250

**Published:** 2025-04-18

**Authors:** Sebastian Bockholt, Georg Gosheger, Burkhard Moellenbeck, Kristian Nikolaus Schneider, Jan Schwarze, Christoph Theil

**Affiliations:** ^1^ Department of Orthopedics and Tumor Orthopedics Muenster University Hospital Muenster Germany

**Keywords:** revision TKA, TKA, total knee arthroplasty, UKA, unicompartmental knee arthroplasty

## Abstract

**Purpose:**

Medial unicompartmental knee arthroplasty (UKA) is a treatment option for medial knee osteoarthritis, with an increase in surgeries over the last few years. However, the results of revision total knee arthroplasty (TKA) after a UKA vary greatly. The purpose of the study was to examine the survival after revision TKA of a failed UKA.

**Methods:**

This is a retrospective single‐centre analysis that includes 35 revision TKA procedures after the failed UKA performed from 2004 to 2019. The median follow‐up after revision TKA was 39 months (interquartile range [IQR]: 32–52). The indication for revision of the UKA was aseptic loosening in 49% of patients (17/35). We evaluated demographic factors, reason for revision and revision implant used with descriptive statistics. Implant survival analysis with a focus on re‐revision‐free survival and potential re‐revisions was performed using Kaplan–Meier survival curves. Differences in survival were analyzed using the log‐rank test. *p* Value was set at 0.05.

**Results:**

Forty per cent (14/35) of revision implants were posterior stabilized revision TKA, followed by 34% (12/35) of condylar constrained designs and 23% of rotating hinged TKA (8/35). Only one patient was revised to a cruciate retaining primary implant (3%). The re‐revision‐free survival after revision TKA amounted to 94% (95% confidence interval [CI]: 91%–100%) after 1 year, 80% (95% CI: 67%–93%) after 2 years and 74% (95% CI: 56%–90%) at 5 years. Twenty‐three per cent of patients (8/35) underwent re‐revision after the initial UKA revision after a median time period of 21 months (IQR: 12–24). The reasons for repeat revision were tibial aseptic loosening in 9% of patients (3/35), periprosthetic joint infection (PJI) in 9% of patients (3/35) and instability in 5% (2/35). Rotating hinge knee implants showed reduced survivorship.

**Conclusions:**

Revision of medial UKA is associated with an increased use of more elaborate and complex revision implants. There is a substantial risk of repeat revision, with aseptic tibial loosening and PJI being the main reasons for the failure of this series.

**Level of Evidence:**

Level III.

**Abbreviations**
ASA: American Society of Anesthesiologists
CCI: Charlson comorbidity index
CI: confidence interval
PJI: periprosthetic joint infection
TKA: total knee arthroplasty
UKA: unicompartmental knee arthroplasty

## PURPOSE

Unicompartmental knee arthroplasty (UKA) is a potential surgical treatment for advanced osteoarthritis of the medial knee compartment [26]. While the indications vary and are evolving [25], most surgeons demand sufficient joint stability and a low extent of degenerative changes in the lateral and patellofemoral joint compartments when performing UKA compared to total knee arthroplasty (TKA). Recent investigations showed a shorter recovery period for UKA compared to TKA in adequately selected patients [9, 10] as well as reduced pain and good improvement in joint function. Considering these findings and the fact that many patients present with medial osteoarthritis, the number of UKAs performed is increasing [27].

Furthermore, the typical complications of TKA apply similarly to UKA [9, 12] but have been reported to be less frequent, at least for some types of complications such as periprosthetic joint infection (PJI) [17, 20, 21]. On the other hand, other complications might be more common, with some reporting a dramatically reduced survival of UKA compared to TKA [5, 16]. However, selection bias and potentially suboptimal indications, as well as patients from inexperienced centres, are potential confounders [6, 25]. Additionally, it is often claimed that conversion to a standard bicondylar TKA is a relatively uncomplicated procedure [11, 23, 25], as the UKA preserves bone stock. This notion somewhat drives the expectation that revising a UKA is a considerably smaller and uncomplicated procedure compared to revision TKA.

In a study from the German arthroplasty registry, Straub et al. [18] were able to demonstrate that among over 36,000 UKAs investigated, the predominant reason for aseptic revision was tibial loosening in 14% of revisions, followed by progression of the arthritis in 8% of revisions. Therefore, one particular issue that also guides the implant choice at the time of revision is the amount of tibial bone loss that can be extensive in revision UKA.

One study on 33 UKAs [3] that were revised for aseptic complications found that only 45% of their revisions were actually revised to a standard TKA. In their study, 55% required the use of more complex implant systems, including stems or augments. These reconstructions can be more challenging and prone to subsequent complications and associated re‐revisions. However, they might be necessary in order to reduce the risk of early repeat revision, particularly for recurrent tibial loosening. One registry study on over 4000 revised UKAs found improved re‐revision‐free survivorship if a tibial stem extension was used either alone or in conjunction with an augment [8].

Furthermore, as UKAs are more often performed in younger individuals [25, 26], the survivorship of a potential revision TKA is of interest due to generally good life expectancy.

This study investigates the outcome of UKA revisions at a single centre, focusing on implant choice at the time of the revision, survivorship of the revision implant and associated re‐revisions. We hypothesize that tibial loosening is a main issue and that more complex revision implants are associated with poor survival.

## METHODS

### Study design

Prior to the study, ethical approval was obtained by the local ethics committee (Ethikkommission der Ärztekammer und der Westfälischen Wilhelms Universität Münster, reference numbers 2018‐123‐f‐S and 2018‐180‐f‐S).

We retrospectively queried our institution's prospectively maintained database on arthroplasty surgeries and identified all revisions of UKAs that were performed between January 2004 and December 2019.

We investigated implant survival of the UKA, reason for revision, choice of the revision implant, implant fixation, revision‐free survivorship and potential re‐revisions.

### Patients

The cohort included 36 patients who underwent revision surgery of a UKA. One patient had a follow‐up of less than 24 months, leaving 35 patients for final analysis. All other patients were followed for a minimum of 2 years. The median follow‐up amounted to 39 months (interquartile range [IQR]: 32–52 months). The median time from primary implantation of the UKA to the first revision surgery was 25 months (25%–75% IQR: 18–45 months).

Women represented 60% of the patients included (21/35). The median age at the time of revision of the UKA was 65 years (IQR: 59–77 years) and the median body mass index (BMI) amounted to 30.1 kg/m^2^. The median Charlson comorbidity index (CCI) was 2 (IQR: 1–4), and the median American Society of Anesthesiologists (ASA) Score was 3 (IQR: 2–3). All patients had their primary medial UKA implanted for osteoarthritis.

### Definitions

We defined prosthetic failure following the revision TKA as requiring revision and exchange of the implant as the primary end‐point. The secondary study end‐point was re‐revision.

The diagnosis of loosening was based on clinical and radiological findings as proposed by the Knee Society's evaluation system with three radiographic views [17]. During the study period, there initially was a protocol of selective aspiration analogue to the Centre for Disease Control criteria based on serum inflammatory markers, while later infection was diagnosed using the criteria of the Musculoskeletal Infection Society of 2011 [3]. After PJI, treatment success was defined based on the modified Delphi consensus criteria [18, 19].

We extracted demographic data from the electronic patient files and calculated the BMI, age‐adjusted CCI [20] and ASA class [21].

### Surgical procedures and implant features

Surgical access was gained through a standard medial peripatellar arthrotomy. For the revision TKA, existing components were carefully removed, and a thorough debridement was performed, removing all scar tissue and, in cases of PJI, all infected tissue. In all revisions, a minimum of three to five microbiological samples were taken and cultured for a minimum period of 7–14 days depending on microbiological growth.

The choice of the implant was made at the discretion of the surgeon, including factors such as patient age, bone quality, knee joint stability, bone defects and the underlying cause for revision. For small defects and stable joints, the Genesis unconstrained prosthesis with a posterior stabilized or deep dished inlay (Smith and Nephew) was used. For ligament instability or the need for additional anchorage, the Genesis‐constrained TKA with condylar‐constrained inlays and the possibility of additional augmentation of bone defects with wedges or stems (Smith and Nephew) was used. For gross instability and extensive defects, a rotating hinge implant (GenuX, Implantcast) was implanted. Stem anchorage usually included short, cemented stems for the Genesis TKA and uncemented diaphyseal engaging stems for the GenuX implant. 6% of patients (2/35) had a tibial porous metal cone implanted (Zimmer, trabecular metal).

### Statistical analysis

Data collection and statistical analysis were performed using Excel (Microsoft Corporation) and SPSS Statistics for Windows Version 25 (IBM Corporation). All patient records were anonymized prior to analysis.

Descriptive statistics were used to analyze the data distribution and means, and ranges were calculated for parametric data, medians and IQRs for nonparametric data. Contingency tables were analyzed using the chi‐square test. While parametric data was analyzed using the Student's *t* test, non‐parametric analyses were performed using the Mann–Whitney *U* test. Survival analysis was performed using the Kaplan–Meier method, and differences in survival and influencing factors were assessed using the log‐rank test. Statistical significance was defined as *p* ≤ 0.05.

## RESULTS

### Implant revisions and choice of implants

The indications for revision of the UKA were aseptic loosening in 49% of patients (17/35), progress of the joint degeneration in 26% (9/35) of patients, PJI in 20% (7/35) of patients and instability in 5% (2/35) of patients. In patients with aseptic loosening, the tibial component only was loose in 15/17 patients while the remaining two patients had loosening of both components.

The choice of implants and fixation strategy are displayed in Tables 1 and 2.

**Table 1:** Choice of constraint at the time of revision TKA.

Variable	% (*n*/*n*)
Posterior stabilized	40 (14/35)
Semiconstrained/condylar constrained	34 (12/35)
Rotating hinge	23 (8/35)
Cruciate retaining	3 (1/35)

Abbreviation: TKA, total knee arthroplasty.

**Table 2:** Implant fixation at the time of revision TKA.

Variable	% (*n*/*n*)
Femoral stem	57 (20/35)
Femoral stem cemented	30 (7/20)
Tibial stem	86 (30/35)
Tibial stem cemented	47 (14/30)
Tibial cone	2 (6/35)

Abbreviation: TKA, total knee arthroplasty.

### Implant survival and re‐revisions

The re‐revision‐free survival after revision of the UKA to a TKA amounted to 94% (95% CI: 91%–100%) after 1 year, 80% (95% CI: 67%–93%) after 2 years and 74% (95% CI: 56%–90%) at 5 years.

Twenty‐three per cent of patients (8/35) underwent re‐revision after this initial UKA revision after a median time period of 21 months (IQR: 12–24). The reasons for repeat revision were aseptic tibial loosening in 9% of patients (3/35), PJI in 9% of patients (3/35) and instability in 5% (2/35).

All cases of aseptic loosening were on the tibial side.

### Risk factor analysis regarding re‐revision

The type of implant used did significantly impact the risk of re‐revision: Posterior stabilized and condylar constrained revision TKA had a significantly (*p* < 0.001) better survival compared to cruciate retaining revision TKA without stems or augments. However, this effect is fragile and based on only one patient with a CR TKA at the time of revision.

Furthermore, patients who underwent revision TKA using a rotating hinge TKA had worse survival compared to posterior stabilized and condylar constrained revision TKA (63% vs. 93% vs. 83%) after 2 years (Table 3). This difference was significant (*p* < 0.001) for PS implants and there was a trend (*p* = 0.075) for condylar‐constrained implants. The dominant mode of failure in the rotating hinge revision TKA group was (re‐)infection, which occurred in 38% of patients (3/8) after a median time of 11 months. One of these patients had re‐reinfection with a different organism.

**Table 3:** Re‐revision‐free survival depending on the type of implant used during the first revision.

Implant type	Re‐revision‐free survival after 2 years (95% CI)
Cruciate retaining	0%
Posterior stabilized	93% (80%–100%)
Condylar constrained	84% (70%–90%)
Rotating hinge	63% (33%–93%)

Abbreviation: CI, confidence interval.

However, the risk of re‐revision did not differ between men and women after 2 years (71% [95% CI: 47%–95%] vs. 86% [95% CI: 70%–100%], *p* = 0.181). Additionally, there was no difference in the risk for re‐revision depending on the indication of the first revision (*p* = 0.625).

## DISCUSSION

The most important findings of the study were that the main reason for the initial revision surgery was aseptic tibial loosening followed by the progression of osteoarthritis. The re‐revision‐free survivorship was 80% (95% CI: 67%–93%) after 2 years, with 23% of patients undergoing re‐revision mainly for repeat tibial loosening and PJI. With respect to the choice of implant, we found that rotating hinge implants showed worse survival compared to condylar constrained and posterior stabilized implants, and almost 90% of patients had a tibial stem implanted. These findings highlight the potential spectrum of complications in this seemingly rather uncomplicated revision situation. Particularly repeat tibial loosening appeared to be a major issue in this cohort. Surgeons who perform UKA revisions, particularly for tibial loosening, should potentially employ more elaborate methods of fixation, such as cones and sleeves, rather aggressively in order to mitigate the revision risk for this indication. However, further studies are needed considering this study's retrospective design and limited numbers.

The main indication for revision UKA in this study was aseptic tibial loosening, which was the reason for revision in 43% of patients. The indications for revision of UKA have been studied previously: One large review article [22] investigated the reasons for revision of UKA, including 24 cohort studies and three registry‐based studies, including a total of 750 failed UKAs based on cohort studies and over 8000 failed UKAs from registry studies. While there were differences between registry and cohort studies in terms of fewer revisions for aseptic loosening in the cohort studies, aseptic loosening was a leading cause (36% of revisions) based on the registry data and the second most common reason in the cohort studies included (24% of revisions). This is similar to the present study, although it is noteworthy that the percentage of loosening in this study is relatively high considering the short period after the initial UKA. Tay et al. [22], in a review, found that aseptic loosening was a typical early‐mid to mid‐term and late follow‐up complication. However, the definitions and follow‐up periods vary among studies. While it has not been uniformly reported if aseptic loosening predominantly affected the tibia, it appears intuitive to assume this as tibial loosening is more common [14].

Based on our findings, patients with a medial UKA should be counselled regarding the relative risk of tibial loosening. This issue is supported by a study from the Norwegian arthroplasty registry [5], which found an improvement in TKA survival over time while the revision risk of UKA for various reasons, including aseptic tibial loosening, was constantly high and overall greater than the risk of revision TKA. Therefore, the authors argue that the potential benefits of performing UKA versus TKA should be balanced with the specific risk of revision.

At the time of revision of a UKA, surgeons are confronted with the optimal choice of a revision implant [13]. While this is obviously a multi‐factorial decision based on the indication for revision, bone defects or deformity present, as well as patient factors such as age or certain comorbidities, revision of a UKA has, however, been considered a less complicated procedure leading to a relatively lower threshold to perform revision [7]. However, it is accepted that the use of revision TKA implants at the time of revision of a UKA is high [2, 3]. One study on 49 patients undergoing revision from UKA to TKA found that 34% of patients required a revision TKA implant [4]. They predominantly used stems and augments in this cohort, although constrained implants were rarely required (8%) [4]. This finding is supported by a meta‐analysis of 341 patients from five cohort studies on the implant choice for revising a UKA to a TKA [19]. The authors found that polyethylene thickness was significantly higher, and the use of stems, augments and bone grafts increased drastically. This is comparable to the findings in the present study that found that 85% of revision TKAs following the UKA required a tibial stem and 57% required a femoral stem.

Furthermore, the risk of re‐revision might be lower if tibial stems and augments are used based on data from the Australian arthroplasty registry [8]. On the other hand, the use of constrained and even hinged implants (57% of patients in this cohort) was more common compared to a study on 193 UKAs that underwent revision at a single academic institution [11] that did not use constrained or hinged implants in any of their cases. While the authors acknowledge that constrained implants might be necessary if there is incompetence, particularly in the medial collateral ligament, this apparently was not the case in their series. While the choice of the constraint depends on various factors, including patient factors and, to some extent, surgeon preference, we hypothesize that the higher percentage of patients treated for PJI in our cohort (20% vs. 2.6%) with the need for aggressive debridement might explain that difference. Considering these findings, surgeons should counsel patients with a UKA that revising this implant to a TKA might not be an ‘uncomplicated’ conversion. From the perspective of implant choice, these procedures must be considered a revision TKA with the need to have sufficient revision implants (particularly on the tibial side) available. There appears to be a necessity of utilizing stems and augments quite frequently although the need for additional constraint might vary depending on the indication.

While the risk of revision of a UKA is quite well understood, there are relatively few studies that investigate the risk of re‐revision in this patient cohort. Considering that many UKAs are performed in rather younger patients compared to TKA [5], the risk of re‐revision is of growing importance in this patient cohort. We found that there was a 2‐year re‐revision risk of around 20%, with 23% of patients requiring additional surgery by this time. The reasons were recurrent tibial loosening and PJI in 9% each. One study on 175 UKA revisions found that 13% had a complication, and a re‐revision risk of 4.5% was reported at mid‐term follow‐up. In their series, similar to the present series, the main reason for re‐revision was loosening, which occurred in 5% of patients. However, compared to the present study, there was only a very small percentage of patients undergoing revision with a constraint or hinged implant (6% vs. 57%) as well as a small percentage of patients treated for PJI (3% vs. 20%). The use of hinged implants, as well as more constraint, was associated with a higher risk of re‐revision in the present study, which might explain the differences.

While the use of rotating hinge implants might become necessary in complicated situations with gross instability, severe bone loss or following surgery for PJI [1, 15, 24, 28], surgeons must consider the increased risk of recurrent revision. This study found (recurrent) PJI to be the main reason for re‐revision in patients with a rotating hinge implant that was significantly higher compared to less‐constrained implants.

This study provides some novel findings regarding a potentially increased risk of re‐revision and relatively poor survivorship for revised UKAs at a single centre. This should caution surgeons to downplay the revision of a UKA as an uncomplicated conversion surgery. However, the present study has several limitations: First, it is a retrospective study that relies on the completeness of follow‐up and is prone to recall and selection bias. The actual risk of re‐revision might be even higher. Furthermore, while we focused on the indication, implant choice and re‐revision risk as the main outcome parameters, unfortunately, no routine functional data are available. Therefore, it is unclear how patients did in the common outcome measures following these procedures. This should be addressed in future studies, particularly focusing on re‐revisions and the potential effect on patient‐reported functional outcomes.

## CONCLUSIONS

Revision of medial UKA is associated with an increased use of more elaborate and complex revision implants. There is a substantial risk of repeat revision, with aseptic tibial loosening and PJI being the main reasons for the failure of this series.

## AUTHOR CONTRIBUTIONS

**Sebastian Bockholt**: Conceptualization; writing—review and editing; resources; validation. **Georg Gosheger**: Conceptualization; resources; writing—review and editing. **Christoph Theil**: Conceptualization; methodology; investigation; data curation; formal analysis; writing—original draft preparation; writing—review and editing. **Burkhard Moellenbeck**: Methodology; validation; investigation; data curation; formal analysis; writing—review and editing. **Jan Schwarze**: Validation; investigation; formal analysis; writing—review and editing. **Kristian Nikolaus Schneider**: Validation; investigation; formal analysis; writing—review and editing. All authors have read and agreed to the published version of the manuscript.

## CONFLICT OF INTEREST STATEMENT

The authors declare no conflicts of interest.

## ETHICS STATEMENT

The study was conducted in accordance with the Declaration of Helsinki and approved by the Institutional Review Board (or Ethics Committee) of Ethikkommission der Ärztekammer und der Westfälischen Wilhelms Universität Münster, reference numbers 2018‐123‐f‐S and 2018‐180‐f‐S. Informed consent was not deemed necessary because this is a study that retrospectively analyzes routine patient data.

## Data Availability

Raw data are available upon reasonable request to the corresponding author.
